# Deep learning diagnostic and severity-stratification for interstitial lung diseases and chronic obstructive pulmonary disease in digital lung auscultations and ultrasonography: clinical protocol for an observational case–control study

**DOI:** 10.1186/s12890-022-02255-w

**Published:** 2023-06-02

**Authors:** Johan N. Siebert, Mary-Anne Hartley, Delphine S. Courvoisier, Marlène Salamin, Laura Robotham, Jonathan Doenz, Constance Barazzone-Argiroffo, Alain Gervaix, Pierre-Olivier Bridevaux

**Affiliations:** 1 Division of Paediatric Emergency Medicine, Department of Women, Child and Adolescent, Geneva University Hospitals, 47 Avenue de la Roseraie, 1211 Geneva 14, Switzerland; 2 Machine Learning and Optimization (MLO) Laboratory, Swiss Federal Institute of Technology (EPFL), Lausanne, Switzerland; 3 Quality of Care Unit, Geneva University Hospitals, Geneva, Switzerland; 4 Division of Pulmonology, Hospital of Valais, Sion, Switzerland; 5 Division of Paediatric Pulmonology, Department of Women, Child and Adolescent, Geneva University Hospitals, Geneva, Switzerland; 6 Faculty of Medicine, University of Geneva, Geneva, Switzerland

**Keywords:** Lung diseases, Interstitial, Idiopathic pulmonary fibrosis, Idiopathic interstitial pneumonias, Pulmonary disease, Chronic obstructive, Deep learning, Artificial intelligence, Respiratory sounds, Auscultation, Ultrasonography

## Abstract

**Background:**

Interstitial lung diseases (ILD), such as idiopathic pulmonary fibrosis (IPF) and non-specific interstitial pneumonia (NSIP), and chronic obstructive pulmonary disease (COPD) are severe, progressive pulmonary disorders with a poor prognosis. Prompt and accurate diagnosis is important to enable patients to receive appropriate care at the earliest possible stage to delay disease progression and prolong survival. Artificial intelligence-assisted lung auscultation and ultrasound (LUS) could constitute an alternative to conventional, subjective, operator-related methods for the accurate and earlier diagnosis of these diseases. This protocol describes the standardised collection of digitally-acquired lung sounds and LUS images of adult outpatients with IPF, NSIP or COPD and a deep learning diagnostic and severity-stratification approach.

**Methods:**

A total of 120 consecutive patients (≥ 18 years) meeting international criteria for IPF, NSIP or COPD and 40 age-matched controls will be recruited in a Swiss pulmonology outpatient clinic, starting from August 2022. At inclusion, demographic and clinical data will be collected. Lung auscultation will be recorded with a digital stethoscope at 10 thoracic sites in each patient and LUS images using a standard point-of-care device will be acquired at the same sites. A deep learning algorithm (*DeepBreath*) using convolutional neural networks, long short-term memory models, and transformer architectures will be trained on these audio recordings and LUS images to derive an automated diagnostic tool. The primary outcome is the diagnosis of ILD versus control subjects or COPD. Secondary outcomes are the clinical, functional and radiological characteristics of IPF, NSIP and COPD diagnosis. Quality of life will be measured with dedicated questionnaires. Based on previous work to distinguish normal and pathological lung sounds, we estimate to achieve convergence with an area under the receiver operating characteristic curve of > 80% using 40 patients in each category, yielding a sample size calculation of 80 ILD (40 IPF, 40 NSIP), 40 COPD, and 40 controls.

**Discussion:**

This approach has a broad potential to better guide care management by exploring the synergistic value of several point-of-care-tests for the automated detection and differential diagnosis of ILD and COPD and to estimate severity.

*Trial registration* Registration: August 8, 2022. ClinicalTrials.gov Identifier: NCT05318599.

**Supplementary Information:**

The online version contains supplementary material available at 10.1186/s12890-022-02255-w.

## Background

Interstitial lung disease (ILD) encompasses several pulmonary conditions defined by an alteration of the pulmonary interstitium, a restrictive pattern of lung function, and fibrotic scarring on chest computed tomography (CT). Approximately one-third of these disorders have known endogenous or exogenous causes, including environmental or occupational factors, infections, drugs and radiation. Two-thirds are of idiopathic aetiology [1] and comprise a range of subcategories, the most common of which is idiopathic interstitial pneumonia (IIP). In turn, IIP comprises a range of sub-pathologies, such as idiopathic pulmonary fibrosis (IPF) and non-specific interstitial pneumonia (NSIP) [2]. Identifying patients with IIP at the earliest possible stage is essential for care management as treatment is aimed at slowing the irreversibly debilitating and ultimately fatal progression. Delay in specialist referral is associated with a higher mortality, irrespective of disease severity [3]. With a mean delay of 2.2 years between the onset of symptoms and specialist referral, the investigation of competing diagnoses by non-specialist providers can be costly for both patients and healthcare providers [3]. However, given the initial non-specific symptomatic presentation, the need for advanced diagnostic tools, such as high-resolution chest CT (HRCT), and an expertise in the early-stage diagnosis of IPF and NSIP remain desirable and achievable objectives [4]. Distinguishing between IPF and NSIP raises considerable diagnostic challenges as their clinical presentations share many overlapping features. However, the distinction is useful as their response to treatment differs markedly [5]. Until now, with limited treatment options benefiting mostly patients in the early stages of the disease [6, 7], many patients will progress towards disability or death [8]. Despite research and advances in therapy, ILDs remain a worldwide health challenge affecting millions of people each year [9], emphasizing the need to make progress in diagnosis and prevention.

Different measures have been proposed to improve the early diagnosis of ILDs [10, 11]. In particular, the identification of the so-called “velcro”-like crackles on lung auscultation by primary care doctors has been suggested as an early and strongly predictive sign of IPF or fibrotic NSIP [4, 12]. For instance, while IPF and NSIP typically have fine velcro-like crackles audible on the mid-to-late inspiratory cycle, chronic obstructive pulmonary disease (COPD) tends to have coarse crackles occurring during the early inspiratory cycle [13]. As stethoscopes are readily available, inexpensive and non-invasive, they constitute an adequate tool to detect velcro-like crackles in the early stages of IPF or fibrotic NSIP to shorten the diagnostic delay and allow the prompt referral to specialised care. However, conventional auscultation is a highly subjective skill limited by inter-listener variability and human perceptual ability to distinguish between lung sounds and their temporal occurrence in the respiratory cycle. Inherent heterogeneity in stethoscope quality, background noise and patient-related factors, such as obesity or chest deformities, are other limiting factors. To overcome these drawbacks, research efforts have been devoted to improve computerised respiratory sound recording with electronic stethoscopes and an objective analysis based on advanced digital acoustic signal processing [14–17]. The advent of deep learning in recent years took the analysis of auscultation signals one step further by allowing an enhanced detection of abnormal lung sounds in patients with respiratory diseases [18–20].

Several studies have assessed the broad adoption and impact of deep learning to help diagnose COPD [21–23], the third leading cause of death worldwide [24]. The vast majority developed predictive models to cover a wide range of objectives, the main ones being the diagnosis and severity classification of the disease [25, 26]. A 2022 review of artificial intelligence (AI) techniques in COPD yielded 156 articles relevant to the application of AI in COPD research, including 56 concerning diagnosis, 65 on its prognosis, 54 on COPD severity classification, and 17 on the management of the disease [27]. Most studies have used a variety of features, including patient physiological characteristics, comorbidities, symptoms, vital signs, biomarkers, genomic information, pulmonary function tests, CT images, hospitalization information, and/or breath sounds [28, 29]. Regardless of the method(s) chosen, COPD remains an incurable and progressive disease and diagnosis at the early risk stage is important. In this sense, the work of Altan et al. is innovative. The deep learning algorithms they used on analysing multiple lung auscultation points for the early diagnosis of COPD achieved high classification performance rates [30, 31]. Achieving this with a method as conventional as lung auscultation can reduce the need for additional, more extensive, time-consuming, expensive or invasive diagnostic tests.

Conversely, research regarding IPF and NSIP are scarce and have focused mostly on datasets collected through radiological [32–34], genomic [35, 36] or functional tests [37]. Pancaldi et al. described the use of an AI algorithm to detect the presence of velcro-like crackles in patients with rheumatoid arthritis and a suspicion of ILD [17, 38]. However, to our knowledge, no study has investigated the benefit of deep learning-aided diagnostic tools for early IPF and NSIP diagnosis using respiratory sound analysis in adults. This might allow doctors to assess acoustic signatures more objectively and thus allow a more standardised and potentially earlier diagnosis in patients presenting at primary care clinics with non-specific, chronic respiratory symptoms. On the other hand, lung ultrasound (LUS) is already the standard of care for detecting consolidations, diagnosing pneumonia and guiding pleural taps. The distinction between A (normal aeration) and B (alveolar-interstitial syndrome) lines on LUS is clinically important and forms the backbone of multiple clinical decision trees for real-time respiratory diagnoses and treatment choices [39]. As such, not only is LUS a relevant gold standard for lung pathology, but it could also benefit from automation by deep learning.

We developed a series of deep learning algorithms on digital lung auscultation (*DeepBreath*) and LUS to detect a range of physiological and pathological lung diseases, including (COVID-19) [40]. This study will seek to explore the synergistic value of several point-of-care-tests for the AI-aided detection and differential diagnosis of ILD and COPD, as well as estimate of severity, with the aim to better guide and improve care management in adults.

## Methods

### Study design

**Fig. 1:**
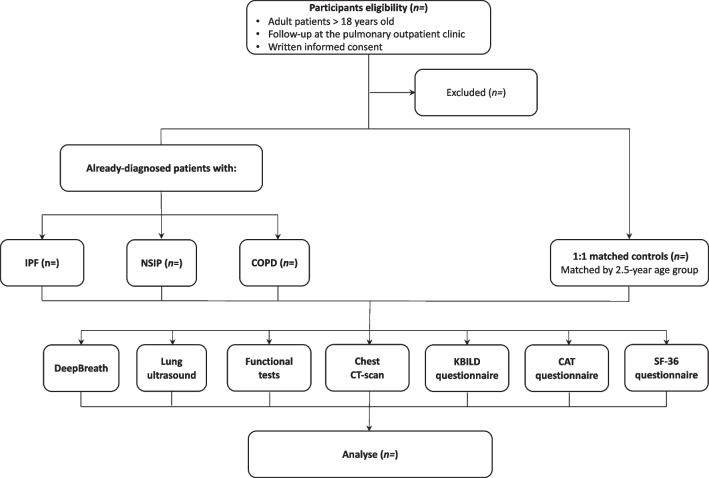
Study flowchart

This is a single-centre, prospective, population-based, case–control study that will be carried out in subjects with IPF, NSIP and COPD within a pulmonology outpatient clinic in Switzerland, with a total of approximately 7000 specialised consultations per year. Figure 1 shows the study flowchart and Table 1 details the study schedule. The present study protocol adheres to the Strengthening the Reporting of Observational studies in Epidemiology (STROBE) Statement [41].

**Table 1:** Study schedule

	Study period		
	Pre-enrolment	Enrolment	Close-out
Timepoint	−*t*_1_	*t*_0_	
Study procedures			
Recruitment		✓	
Eligibility screening		✓	
Informed consent form		✓	
Assessments			
Case report form (demographic + clinical data)		✓	✓
EKO CORE digital auscultation collection		✓	✓
Pulmonary function tests		✓	
Chest CT-scan/X-ray	✓		
Lung ultrasound		✓	
K-BILD questionnaire		✓	
CAT questionnaire		✓	
SF-36 questionnaire		✓	

✓: Performed

### Population

Inclusion criteria are consecutive, consenting adult outpatients (> 18 years) with IPF (group 1), NSIP (group 2) and COPD (group 3) already diagnosed prior to the consultation (index) date. Probable and definitive IPF diagnosis will be made according to the Fleischner Society Consensus [42], NSIP diagnosis with the American Thoracic Society classification [2, 43], and COPD with the Global Initiative for Chronic Obstructive Lung Disease criteria [44]. Consenting, age-matched (± 2.5 years) individuals with normal lung function (spirometry, lung volume and transfer factor for carbon monoxide [TL_CO_]) followed in the outpatient clinic with a similar quality of electronic medical records, but for diseases other than the outcome of interest, will serve as the 1:1 control group (group 4). This latter group will comprise patients with obstructive sleep apnoea, follow-up of occupational lung diseases (miners, chemical workers, etc.), and follow-up of pulmonary nodules (considered benign after 2 years). Reasons for pulmonary follow-up among the controls will be reviewed and reported in a supplementary file. Identifying all cases with the outcome of interest and selecting controls for comparison is a more efficient and resource-sparing study design than a full cohort study. Exclusion criteria are: (1) patients who cannot be mobilised for posterior auscultation; (2) those known for severe cardiovascular disease with pulmonary repercussions; (3) patients known for a concurrent, acute, infectious pulmonary disease (e.g., pneumonia, bronchitis); (4) patients known for asthma exclusive from COPD; (5) patients with alpha-1-antitrypsin deficit; (6) a physical inability to follow procedures; and 7) inability to give informed consent.

### Recruitment and informed consent procedure

Patients will be recruited from an outpatient pulmonology clinic in Switzerland in daily clinical practice. Participants will provide written informed consent, provided that they have had sufficient time for consideration and the opportunity to ask questions. Important concepts will be highlighted via bulleted text. A checkbox will assess whether participants understand key consent information in the presence of study investigators. These consent forms will be collected and countersigned by the study investigators and stored securely in an access-controlled room. No financial compensation will be offered to participants.

We anticipate that withdrawal and discontinuation will be limited as the study offers the advantage of taking place in a single centre and during a single, short (i.e., 60 min) intervention period on the day of a routine clinical visit. In the case of withdrawal after informed consent, the individual’s data collected so far and related to the intervention will be destroyed/deleted. Any withdrawal and/or discontinuation will be justified and reported in final publications in anonymised form.

### Hypothesis and objectives

#### Primary hypothesis

We hypothesise that point-of-care digital lung auscultation and LUS have a clinically exploitable predictive performance for the detection of pathological acoustic and sonographic signatures in patients with ILD. Furthermore, we propose that these signatures are sufficiently unique to not only discriminate ILD patients from control subjects, but also from COPD and other respiratory diseases, and perhaps even to categorise the various severity grades and subtypes of ILD, when determined. We further hypothesise that the automated interpretation of lung auscultation and LUS by deep learning could match or outperform expert evaluation and standardise lung auscultation and LUS interpretation.

#### Primary objective

To collect a systematic sound bank of digital lung auscultation and images for the development of deep learning algorithms that predict pathological signatures of ILD in an adult population to: (1) discriminate ILD from non-ILD lung sounds and images; (2) predict ILD clinical severity; (3) differentiate ILD from COPD; and (4) possibly determine the subcategories of ILD (i.e., IPF versus NSIP).

#### Secondary hypothesis

International clinical practice guidelines recommend to suspect IPF and NSIP in the presence of velcro-like crackles [45] (and similarly for coarse crackles in COPD). However, there are few data indicating whether these sounds are associated with clinical, functional, and radiological characteristics upon ILD diagnosis [4, 46].

#### Secondary objective

To investigate whether velcro-like crackles labelled by human experts are associated with the aforementioned characteristics in patients with IPF and NSIP (and similarly for coarse crackles and COPD). The impact of the diseases on patients’ health-related quality of life will be measured with standardised questionnaires.

Our overall hypothesis is that the use of *DeepBreath* might substantially improve the early and accurate diagnosis of patients with chronic lung disease.

### Primary and secondary outcomes

The primary outcome is the diagnostic of ILD, both IPF and NSIP, versus control subjects or COPD. We will assess the predictive performance of the *DeepBreath* algorithm-evaluated lung auscultation and LUS in the following identification and risk stratification tasks as follows: (1) to discriminate ILD from control subjects (according to expert clinical diagnosis [42]); (2) to differentiate ILD from COPD; (3) to predict ILD clinical severity (according to a HRCT grading scale^1^ and lung function tests^2^); and (4) to differentiate the subcategories of ILD (such as IPF, NSIP) according to the gold standard diagnosis [2, 43, 44]).

Secondary outcomes are the clinical, functional and radiological characteristics of IPF, NSIP and COPD diagnosis. We will: (1) compare the predictive performance of human, expert-identified acoustic and LUS signatures in the above predictive tasks (Kappa coefficient); (2) assess diagnostic performance of a model trained to detect crackles; (3) explore the utility of adding clinical data (signs, symptoms, demographics, medical history and basic paraclinical tests) to the breath sound algorithms; and (4) determine the impact of the diseases on subjects’ health-related quality of life measured with the standardised King’s Brief Interstitial Lung Disease (K-BILD) [47], the COPD assessment test (CAT) [48], and the 36-item Medical Outcomes Study Short-Form Health Survey (SF-36) [49] severity assessment questionnaires.

### Study procedure

The study will be performed over a period of 6 months. Recruitment can be stopped before the anticipated end if the inclusion of 160 patients is reached before. A trained research nurse/doctor (MS/LR) will recruit the subjects during a single routine consultation in the outpatient clinic. This will include checking the selection criteria for each patient prior to study participation, obtaining written informed consent, administering questionnaires on demographic characteristics (age, sex, occupation, long-term exposure to occupational or environmental agents, etc.), relevant medical history, and symptomatic presentation (Additional file 1), which will be captured by an electronic case report form to be completed by the study coordinator on a tablet. The nurse/doctor will also administer the standardised K-BILD [47], CAT [48], and SF-36 [49] severity assessment questionnaires, and record lung sounds during 5–7 min with an electronic stethoscope in the same zones as LUS acquisition, as previously proposed by our group [40]. For the LUS examination using a standard point-of-care ultrasound device, an adapted version of our previous 10-point acquisition protocol [50] will be used, which involves scanning the anterior superior, anterior inferior, posterior superior, posterior inferior and lateral thorax regions. In addition, pulmonary functional tests (conducted with patients in a stable condition) will be collected, as well as chest X-rays with two incidences (posterior-anterior and lateral) and HRCT scans for IIP patients (within 12 months). Controls will not be exposed to a chest X-ray and/or CT scan unless required as part of their routine follow-up; cases will have undergone such imaging given that it is part of their diagnostic evaluation.

All LUS images and lung sounds captured will be digitally recorded and transferred via a secured internet connection together with relevant metadata to a secure server. For study quality control purposes, the quality of the image and the interpretation of a random sample of images will be evaluated retrospectively by an experienced radiologist. The images will be further used for secondary studies developing machine learning algorithms and AI for LUS diagnosis. As this study will take place during outpatient visits under usual conditions and with conventional diagnostic measurement tools, we do not expect any problems that would put participants at a greater risk than normal exposure in daily clinical practice.

#### Lung sound recording

The frequency range of normal lung sounds extends from below 100 Hz to 1000 Hz, with a sharp drop at approximately 100 to 200 Hz [51], whereas tracheal sound extends between 100 to 5000 Hz. In the lower band range (under 100 Hz), heart and thoracic muscle sounds overlap. Abnormal lung sounds (wheezing, rhonchi etc.) have characteristic frequencies and duration, differentiating them from each other [51]. In particular, fine velcro-like crackles are caused by explosive openings of the small airways, have a distinguishable high-pitched frequency of about 650 Hz, and a typical short duration of about 5 ms.

In this study, the lung sounds will be gathered digitally in all subjects with the same Eko CORE digital stethoscope (Eko Devices, Inc., CA, USA). Four anterior thoracic sites (superior and inferior bilaterally), 4 posterior sites (superior and inferior bilaterally),) and 2 lateral sites (right, left) will be auscultated per patient using the stethoscope. For each auscultation site, a 30-s digital recording will be acquired. Patients will be informed of the necessity to breathe deeply. All signals will be saved as 16-bit resolution, 4 kHZ-sampled WAV files. The built-in filter will range from 20 to 2000 Hz. Heart and thoracic muscle sounds, as well as other background low-frequency noises, will be filtered out through EKO software’s high-pass filters. Coded recorded sounds will be synced in real-time to a General Data Protection Regulation (GDPR)-compliant secured cloud-storage location. Random auscultatory recordings will be reviewed by the study investigators for quality control.

#### LUS

LUS is a well-established, consumable-free and non-invasive point-of-care respiratory examination. While it is less ubiquitous than the stethoscope, its new portable and affordable ultrasound-on-a-chip design, pluggable into a mobile device, has the potential to be integrated into the standard clinical examination without incurring extra costs, time, radiation or specialist consultation. It has been shown to be highly effective in detecting lung consolidation in pneumonia [52]. For COVID-19, its diagnostic accuracy matches that of chest CT [53] and it was previously demonstrated that it has an excellent performance for risk-stratification [50]. LUS has been found to be very sensitive to detect subtle changes in the subpleural space. Fibrosis presents as diffuse, multiple B-lines where thickening or irregularity of the pleural line is associated with scarring and disease advancement. Disease severity is also seen in the total number of B-lines, while the average distance between two adjacent B-lines is an indicator of a particular pattern of fibrosis (e.g., pure reticular fibrosis as in IPF compared with the predominant ground glass pattern seen in fibrotic, nonspecific, interstitial fibrosis). The anatomic distribution of these anomalies may also have some relevance to fibrosis type.

In this study, a trained doctor (LR) will perform all LUS at inclusion. Acquisition will be standardised according to protocol [50]. Two images (sagittal and transverse) and 5-s video clips will be systematically recorded for each of the 10 thoracic sites with a Butterfly IQ (Butterfly Network, Guilford, CT, USA), using the lung preset. Reporting of pathological LUS features will be standardised. For every zone, the following patterns will be reported: (1) normal appearance (A lines, < 3 B lines); (2) pathologic B lines (≥ 3 B lines); (3) confluent B lines; (4) thickening of the pleura with pleural line irregularities (subpleural consolidation < 1 cm); (5) consolidation (≥ 1 cm); (6) presence of subpleural nodules; (7) presence of pleural effusion; (8) diaphragmatic excursion (in mm); and (9) diaphragmatic thickening (in mm). The LUS score, used as a correlate of loss of lung tissue aeration, as well as a normalised LUS score (nLUS score) corrected for the number of examined zones, will be calculated in every patient [54].

## AI algorithms

### Diagnostic and risk stratification algorithms

**Fig. 2:**
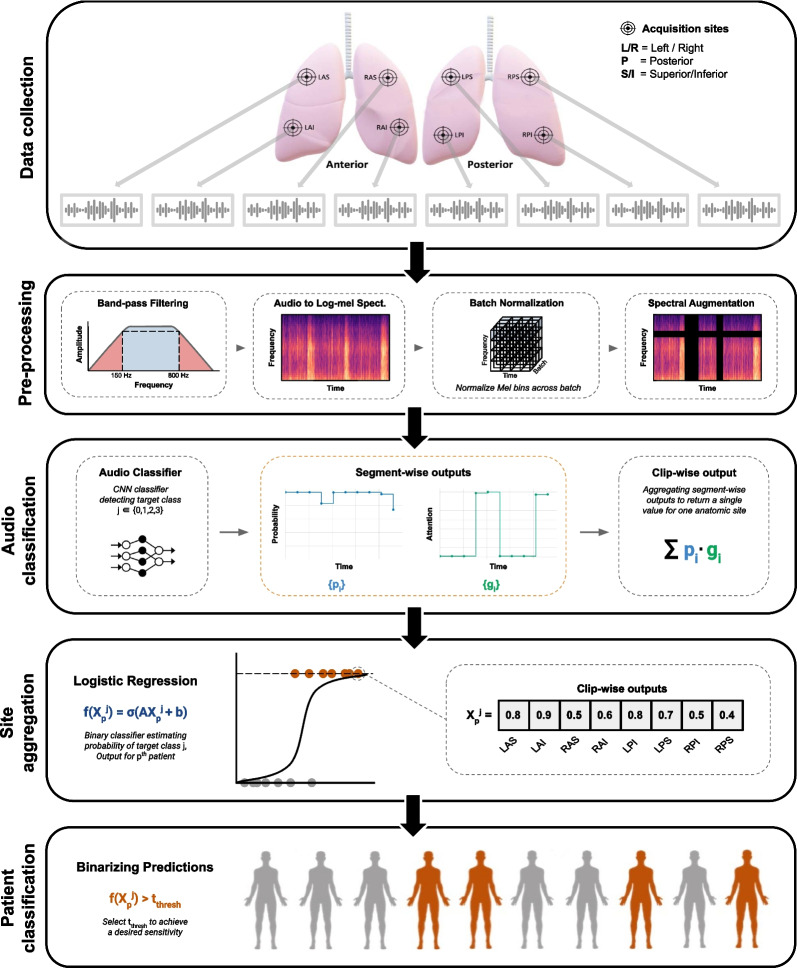
Overview of the DeepBreath binary classification model. Top to bottom: Data collection. Every patient will have 10 lung audio recordings corresponding to 1 per 10 anatomical sites (LAS, RAS: Left and Right Anterior Superior; LAI, RAI: Left and Right Anterior Inferior; LPS, RPS: Left and Right Posterior Superior; LPI, RPI: Left and Right Posterior Inferior; Left and Right Lateral [not shown on the figure]). Pre-processing. A band-pass filter is applied to clips before transformation to log-mel spectrograms which are batch-normalised and augmented and then fed into an audio classifier. Here, a CNN outputs both segment-level prediction and attention values which are aggregated into a single clip-wise output for each site. These are then aggregated by concatenation to obtain a feature vector of size for every patient, which is evaluated by a logistic regression. Finally, patient-level classification is performed by thresholding to get a binary output. The segment-wise outputs of the audio classifier are extracted for further analysis. Used with permission from Heitmann et al. (10.1038/s41746-023-00838-3, Nature Digital Medicine) (Swiss Federal Institute of Technology EPFL, Lausanne, Switzerland)

We will develop *DeepBreath*, a deep learning algorithm to detect the acoustic signatures of IPF, NSIP and COPD from lung sounds. While several state-of-the-art approaches will be tested, the general framework is summarised in Fig. 2. Digital lung auscultations will first be cleaned to crop non-biological frequencies and amplitudes generated by ambient noise not filtered by the stethoscope’s active noise cancelling. The sounds will then be divided into overlapping time windows of between 1 and 10 s and transformed to Mel Frequency Cepstral Coefficients (MFCCs). Several data augmentation techniques will be explored, such as amplitude scaling, pitch shift and random time shift. The effect of each pre-processing method will be tested and the best performing approach according to sensitivity and specificity will be reported. This dataset will then be fed into various deep learning networks (such as convolutional neural networks, Long Short-Term Memory models [LSTM], Transformer architectures, etc.). A prediction on each segment will then be aggregated to represent a patient (including all anatomic sites) and binary classification into positive *vs* negative for diagnostic results will be performed for ILD or control subjects, ILD or COPD, and (if ILD-positive) IPF or NSIP. The same prediction will also be made using LUS images. Risk stratification will use multiclass or regression according to scales obtained from clinical interpretation of LUS, lung function tests, HRCT imagery, K-BILD or CAT, and SF-36 quality of life questionnaires.

### Exploring the synergy of clinical data with breath sounds

Clinical data will be explored for its predictive capacity in the above tasks and added to the breath sound analysis either as a support vector machine (SVM) or in conditional feature extraction upstream of the neural network.

## Clinical assessment of lung auscultation and LUS

The following data will be reviewed by external experts and interpreted using standardised report forms noting the binary presence/absence of several anomalies as well as a text field for other notable observations: routine chest X-ray films (usually 2 incidences, posterior-anterior and lateral); lung auscultation audio clips (10 anatomic regions represented, 30 s recordings of each region); and LUS images (10 anatomic regions represented, 5 s video clips of each region). The analysis will be blinded and the assessor will not have any knowledge of the linked clinical data or association between the various imaging modalities (i.e., IDs are scrambled between media sources and chest X-ray images will be reviewed blinded to the patient’s LUS images, auscultation audio, clinical data, etc.). These blinding and standardisation procedures are expected to minimise performer and study management bias respectively.

## Pulmonary function tests and chest CT scan

For all subjects, spirometry, body-plethysmographic parameters (see details above) and lung diffusion capacity for carbon monoxide (TLco/Kco) will be measured. Participants’ lung images recorded from previous HRCT (or X-rays) during past routine visits will be used. No chest CT scans will be performed as part of this study; only lung images of participants previously recorded as part of their regular follow-up or to be performed in this context will be used. The presence on the chest CT scan of honeycombing, traction bronchiectasis, reticulations, ground-glass opacities, and emphysema will be measured for patients with IPF, NSIP or emphysema. The main chest CT scan features of IPF are reported to be basal and peripheral reticulations, traction bronchiectasis, minimal ground glass opacities, and moderate or extensive honeycombing. For NSIP, the CT scan typically demonstrates bilateral lung involvement and invariably some extent of ground-glass opacities, mainly in the lower zones with fibrotic changes, while honeycombing is not a common feature [55]. The presence on the chest CT scan of structural abnormality, such as ≥ 5% emphysema and/or ≥ 15% gas-trapping and/or airway wall thickness ≥ 2.5 mm [56], will be measured for patients with COPD. The protocol assumes normal lung parenchyma in the control group, which will not be exposed to radiation unless controls have already undergone a recent (i.e., < 5 years) chest CT scan for other reasons. This will be taken into consideration as we will investigate the association of lung sounds with radiological characteristics.

## Questionnaires

Demographics including age, sex, ethnicity, environment (smoking status, long-term exposure to occupational or environmental agents, etc.), treatments, presence of chronic respiratory symptoms or repeated lower respiratory tract infectious diseases, as well as a diagnosis of other comorbidities (obesity, immunodeficiency, alpha-1 antitrypsin deficiency, etc.) will be reported in a questionnaire (Additional file 1). Severity of functional limitations according to the New York Heart Association (NYHA) functional classification [57] will be also reported if available.

The impact of IPF and NSIP on subjects’ health-related quality of life will be measured with the standardised K-BILD questionnaire [47] (use with license agreement), which covers 15 questions exploring 3 health dimension scores (psychological, breathlessness and activities, and chest symptoms) using a 7-point Likert response scale (scores range from 0 to 100, a higher score indicating better health status). The impact of COPD will be assessed with the CAT [48] (use with license agreement) that measures eight items: cough; phlegm; chest tightness; breathlessness; limited activities; confidence leaving home; sleeplessness; and energy. Scores range from 0 to 40, with a higher score indicating a more severe impact of COPD on a patient’s life. The SF-36 will also be used to assess the impact of IPF, NSIP or COPD on patients’ quality of life. The investigators will double-check on-site that the questionnaires are fully and accurately completed. Data collection will be carried out using the online REDCap database (REDCap, Vanderbilt University, Nashville, TN, USA; https://www.project-redcap.org/resources/citations/). Conditions or complaints occurring after enrolment will not be considered in the statistical analyses. Current symptoms at enrolment will be registered.

### Sample size calculation

Each patient will provide 10 audio recordings of 30 s. Samples will be considered at the patient level with all 10 recordings. In deep learning, sample size calculation is an intractable problem that is usually discovered through empirical investigation. The number of samples required to reach a certain performance criterion is dependent on the characteristics of the dataset, the diversity and number of the classes, the degree of data augmentation possible, as well as the complexity of the learning algorithm. Thus, sample size calculations cannot rely on the traditional statistical heuristics that are often used in biostatistics. Rather, sample size estimations in deep learning are mostly made by analogy. Evaluating existing knowledge on similar datasets, we find that the expected proportion of velcro-like crackles in IIP patients is nearly 100% [4], whereas the prevalence of coarse crackles is 71% in COPD patients [58]. The exclusivity of these sounds among groups is not known, but overlap is assumed to be minimal and pathological sounds are by definition absent in non-ILD and non-COPD control subjects. Assuming a similar discriminative power compared to a previous work done by our group (personal communication) to distinguish between normal and pathological lung sounds in pneumonia from 80 patients in balanced classes (40 pathological; 40 controls) with 8 auscultation sites of 30 secs each, we estimate using the same number of patients in each class to achieve convergence at above 80% of the area under the receiver operating characteristic curve (AUROC). Thus, we will aim to enrol at least as many patients in each group: 80 ILD (40 IPF, 40 NSIP); 40 COPD; and 40 controls (i.e., known not to have ILD or COPD, and with normal lung function). As the recruitment site would expect 120 ILD patients (40% with IPF; 60% with NSIP) and 100 with COPD over the space of one year, this number is achievable in the time frame of the study (6 months), even with a 70% consent rate.

This sample size is also predicted to be sufficient for deep learning on LUS. Our preliminary results (personal communication) on COVID diagnosis using deep learning achieved 90% AUROC with 150 patients (balanced classes of 75 COVID + and 75 COVID-). As human experts cannot perceive a COVID-specific signature in LUS with high specificity, this is likely a more technically difficult task than distinguishing ILD and COPD from healthy patients. Indeed, there is ample evidence on the visible signs of ILD on LUS [59].

### Statistical analysis plan

For descriptive statistics related to the clinical data collected, all continuous variables will be reported as medians with their interquartile ranges. Binary and categorical variables distribution will be reported as proportions and percentages. To evaluate baseline demographic differences and outcomes differences between the case and control patients, conditional logistic regressions will be used to account for the matched design. Pearson’s and Spearman’s correlation coefficients will be used to assess the relationship between continuous variables normally and non-normally distributed, respectively. For the primary outcome, each task will be quantified using descriptive statistics (i.e., proportion and type of abnormalities), as well as the AUROC, sensitivity, specificity, positive predictive value, negative predictive value, and likelihood ratios (with 95% CIs over a fivefold cross-validation).

The diagnostic accuracy of each echographic sign will be assessed and sensitivity, specificity, positive predictive value, negative predictive value, positive likelihood ratio, and negative likelihood ratio with their 95% CIs will be calculated. To find the combination of echographic signs with the best diagnostic accuracy, we will compare the performance of several multivariable models, such as logistic regression, random forest and neural networks. Performance will be reported on a test set comprising 20% of the data in a tenfold cross-validation with 95% CIs. As a secondary objective, we will aim to compare the predictive performance of human expert-identified acoustic signatures in the above predictive tasks. First, we will describe the expert labels by the percentage of sound labels attributed to each diagnosis. A multivariable logistic regression will be derived using the clinical data and sound labels to estimate the diagnoses, as for the primary objective. A kappa score will be used to assess the concordance between *DeepBreath* and expert diagnosis consolidated into a basic predictive model.

The K-BILD, CAT and SF-36 questionnaires will be analysed with descriptive statistics. Associations between the questionnaires’ sum scores and lung function parameters will be quantified by Spearman’s rank correlation coefficient. We will consider correlations < 0.3 as negligible, ≥ 0.3 to < 0.5 as low, ≥ 0.5 to 0.7 as moderate, and ≥ 0.7 as strong.

Missing data will be reported and padded with zero in the deep learning network and also assessed according to other labels. Features with more than 50% missing values or with a significant bias in missing data fields will be removed and reported. All statistical tests will be two-sided with a type-I error risk of 5%. Data analysis will be carried out using the latest version of R (R Foundation, Vienna, Austria) for descriptive statistics and statistical tests.

## Discussion

Untreated IPF has the worst prognosis among the different forms of ILD, with median survival ranging from 3 to 5 years from diagnosis [60]. Recent studies suggest that if novel anti-fibrotic medications (pirfenidone and nintedanib) are started early, they can slow the rate of lung function decline and prevent IPF exacerbation, thus reducing mortality [6, 7]. Unfortunately, because of the unspecific nature of the symptoms, the early stage of IPF remains underdiagnosed and many patients will progress to advanced disease and may require lung transplantation [8]. By contrast, the prognosis of NSIP is generally better than that of IPF, with a median survival time of more than 9 years. Systemic steroids and immunosuppressive therapy may be attempted to slow or reverse the course of the disease, but non-responsive individuals may also be considered for lung transplantation [8]. When left untreated, NSIP tends to progress toward fibrotic changes and persistent debilitating symptoms. COPD is also a leading cause of disability worldwide. Patients are generally unaware of their condition for years, leading to a significant delay in diagnosis, the application of preventive measures such as a smoking cessation intervention, and potential treatment [61]. Being able to recognise and diagnose these lung diseases earlier is of the utmost clinical importance.

This study will aim to collect a standardised dataset of digital lung auscultations and derive a deep leaning model able to detect the acoustic and sonographic signatures of the presence and severity of IPF, NSIP and COPD. Recent advances in deep learning are promising to support doctors in standardising the detection and interpretation of complex patterns in pulmonary diseases and AI has proven to outperform doctors in discriminating respiratory pathologies via respiratory functional explorations [62], symptoms [63, 64] and/or radiological examinations [34]. To overcome the subjectivity of human auscultation and the discrepancy in auscultation ability between doctors [16], the development of AI algorithms for the analysis of respiratory acoustic signals has been proposed [19, 20]. In order to meet this requirement, many attempts have been made to develop and apply neural networks to automate the detection and classification of various disease-related breath sounds using machine learning and deep learning-based analysis [14, 64–66]. In particular, recent literature reviews have summarized advances in the implementation of respiratory sound-based AI algorithms in the screening, diagnosis, and classification of COPD [26, 65]. Conversely, the current state of knowledge on the computerized analysis of breath sounds in patients with ILD using AI techniques has not been assessed. Table 2 summarizes the published studies most similar to our research.

**Table 2:** Overview on computerized respiratory sound analysis in ILDs using AI techniques

Related works	Sample size	Purpose	Features	Performance	Issues
Aykanat et al. [67]	40 IPF, 211 COPD, and 574 other pulmonary single or mixed conditions, 805 healthy subjects	Binary classification (healthy vs pathological) and 12-class lung disease classification	SVM, k-NN, GB	Performance for binary classification: Acc 88% to 92% Se 85% to 92% Sp 85% to 88% Performance for lung disease classification: Acc 43% to 68% Se 53% to 96%	Severity classification not done. LUS not used
Charleston-Villalobos et al. [68]	19 ILD (12 IPF, 7 extrinsic allergic alveolitis), 8 healthy subjects	Binary classification (healthy subjects vs patients with ILD)	AR model, SNN	Performance evaluation of the neural network: Acc 76.9% to 98.8%, Se 80.4% to 100%, Sp 73.3% to 100%	Severity classification not done. LUS not used
Flietstra et al. [69]	39 IPF, 95 CHF, 123 PN	Binary classification (IPF vs CHF and IPF vs PN)	BPNN, SVM	Acoustic properties of fine crackles of IPF help distinguish them from crackles of CHF and PN	No healthy subjects. Severity classification not done. LUS not used
Fukumitsu et al. [70]	71 ILD (24 honeycombing + , 47 honeycombing-)	Predicting honeycombing on HRCT by the acoustic properties of fine crackles	FFT	Acoustic properties of fine crackles distinguish the honeycombing from the non-honeycombing group	No healthy subjects. Severity classification not done. LUS not used
Horimasu et al. [71]	34 ILD, 8 COPD or asthma, 7 lung tumor, 5 lung nodule, 6 other	Comparisons of machine-learning-based quantification of four types of lung sounds between lung fields with and without ILD in HRCT and chest X-ray	Described in [72]	AUROC 0.855, Acc 75%, Se 76.1%, Sp 73.6%	Severity classification not done. Results suffered from the presence of background noise. LUS not used
Kahya et al. [73]	23 ILD, 28 COPD, 18 healthy	Binary classification (healthy vs pathological)	AR model, k-NN	Acc 71.1%	Severity classification not done. LUS not used
Kim et al. [74]	112 ILD, 211 COPD, 497 other pulmonary conditions, 51 healthy	Binary classification (healthy vs pathological) and three-class (crackles vs wheezes vs rhonchi) classification	CNN	Performance for binary classification: AUROC 0.93 Acc 86.5% Performance for abnormal sounds classification: AUROC 0.92 Acc 85.7%	Severity classification not done. LUS not used
Malmberg et al. [75]	8 fibrosing alveolitis, 8 emphysema, 8 asthma, 8 healthy subjects	Diagnosis agreement between clinical and machine-learning-based classification of lung sounds	FFT, SOM	Kappa for fibrosing alveolitis 0.54	Small number of patients. Severity classification not done. LUS not used
Manfredi et al. [76]	98 CTD patients (42 ILD + , 56 ILD-)	Identifying CTD patients with possible ILD using lung sound-based binary classification (presence vs absence of ILD) compared with chest HRCT	FFT	Acc 82.6%, Se 88.1%, Sp 78.6%	No healthy subjects. Severity classification not done. LUS not used
Manfredi et al. [38]	137 RA patients (59 ILD + , 78 ILD-)	Identifying RA patients with possible ILD using lung sound-based binary classification (presence vs absence of ILD) compared with chest HRCT	FFT	Overall performance: Acc 83.9%, Se 93.2%, Sp 76.9% Performance for velcro-like crackles detection: Acc 67.2%, Se 69.1%, Sp 65.7%	No healthy subjects. Severity classification not done. LUS not used
Messner et al. [77]	7 IPF, 16 healthy subjects	Binary classification (healthy subjects vs patients with IPF)	CRNN	F-score 92.4% Se 85.9%	Small number of patients. Manual crackles labelling required. Severity classification not done. LUS not used
Messner et al. [78]	5 IPF, 10 healthy subjects	Binary classification (healthy subjects vs patients with IPF)	GRNN	F-score 72.1% Se 71.5%	Small number of patients. Manual crackles labelling required. Severity classification not done. LUS not used
Ono et al. [79]	21 IPN, 10 healthy subjects	IPN detectability and severity classification	FFT	Spectral analysis of lung sounds is useful in the diagnosis and evaluation of the severity of IPN	LUS not used
Pancaldi et al. [17]	70 RA patients (27 ILD + , 43 ILD-)	Identifying RA patients with possible ILD using lung sound-based binary classification (presence vs absence of ILD) compared with chest HRCT	FFT	Acc 90%, Se 92.6%, Sp 88.4%	No healthy subjects. Severity classification not done. LUS not used
Santiago-Fuentes et al. [80]	19 ILD (10 IPF, 9 CPFES)	Binary classification (IPF vs CPFES)	AR model, SNN	Performance evaluation of the neural network: Acc 90.7% to 97.3%, Se 91.8% to 98.3%, Sp 87.5% to 96.3%	No healthy subjects. Severity classification not done. LUS not used
Sen et al. [81]	10 obstructive bronchiectasis, 10 ILD, 20 healthy subjects	Binary (healthy vs pathological) and three class (healthy, bronchiectasis and ILD) classifiers	AR model, SVM	Se 85%, Sp 85% Se 100% (three-class classifier)	Small number of patients. Severity classification not done. LUS not used

ACC, accuracy; AR, autoregressive; AUROC, area under the receiver-operating curve; BPNN, backpropagation neural networks; CHF, congestive heart failure; CF, cystic fibrosis; COPD, chronic obstructive pulmonary disease; CPFES, combined pulmonary fibrosis with emphysema syndrome; CTD, connective tissue diseases; CRNN, convolutional recurrent neural network; FFT, fast Fourier transform; GB, Gaussian Bayes; GRNN, gated recurrent neural network; HRCT, high-resolution computed tomography; ILD, interstitial lung disease; IPF, idiopathic pulmonary fibrosis; IPN, interstitial pneumonia; k-NN, k-nearest neighbor; LUS; lung ultrasound; MPNN, message passing neural network; PN, pneumonia; RA, rheumatoid arthritis; Se, sensitivity; SNN, supervised neural network; SOM, self-organizing map; Sp, specificity; SVM, support vector machine

However, there are some notable differences in these studies, which justify the present work. The main ones are the frequent absence of healthy subjects as a control group and the almost unanimous lack of severity classification or joint use of LUS images. As stated by Charleston-Villalobos et al. [68], a comparison with other attempts to diagnose and classify lung sounds is difficult due to the difference in data acquisition, type of classification scheme, lack of gold standards allowing standardization between studies, and their distinct exploratory nature. In particular, a major flaw of most anterior studies aimed at building deep learning models for diagnostic classification from digital lung sounds is the use of publicly available databases, such as the R.A.L.E repository [82] or the International Conference on Biomedical Health Informatics [83]. These databases have inherent acquisition flaws due to heterogeneity in data collection and methods that create systematic biases between the predicted labels on which new algorithms are built. This is then reflected in the results of studies with an exaggerated excellent predictive performance that prevents their evaluation and comparison with each other. On the contrary, in our study, sounds will come from a cohort of patients under standardised and homogeneous recording conditions. It remains to be determined whether an AI algorithm using respiratory sounds and/or LUS analysis can be used as an initial and accurate diagnostic tool for patients with ILDs or COPD. The diagnosis of IPF, NSIP and COPD in early stages may allow practitioners to appropriately recognise exacerbations of a chronic lung disease, whereas patients may initially be diagnosed as having multiple bouts of acute disease (e.g., bronchitis) without this defined diagnosis [61]. Early diagnosis with AI may therefore allow patients to benefit from prevention measures and the allocation of appropriate treatments aiming to reduce the progression to permanent lung damage and improve the overall prognosis in patients presenting at primary care clinics for non-specific chronic respiratory symptoms. As research in this area is scarce, it is anticipated that the results generated from this study will be of great importance and may be sufficient to change and improve pulmonary primary care practice in a vulnerable population by proposing a faster diagnosis.

This study has several limitations. First, the interpretation and data generated by the algorithm at this stage of our research will not be used for diagnostic purposes or treatment decisions. Both of these points will require further dedicated validation studies in clinical contexts. Second, selection bias can occur in case–control studies when control subjects are not truly representative of the population that produces the cases. In this study, both populations will stem from the same source population in a single-centre outpatient clinic, which may suggest more acute symptoms and pathological lung sounds than those encountered in ambulatory care services. Third, since patients with already-diagnosed IPF, NSIP and COPD will be enrolled, we will not be able to confirm whether *DeepBreath* would have detected these patients at earlier stages. Finally, we acknowledge that the sample size is modest, but it appears to be sufficiently powered in the context of a pilot study.

## Conclusion

The *DeepBreath* model could offer a robust, promising and realistic predictive potential of deep learning to be used as a decision support system by health specialists to better guide clinical care management by exploring the synergistic value of digital lung auscultation and ultrasonography for the automated detection and differential diagnosis of ILD and COPD and to estimate severity. This could be the next frontier in the early diagnosis of COPD and ILD to help improve patient outcomes and quality of life. Furthermore, this study may pave the way for future research based on non-invasive AI models combining point-of-care techniques already commonly used in clinical practice for application to other pulmonary pathologies or even to decentralised care in low-resource settings.

## Supplementary Information

**Additional file 1**. Self-administered questionnaire on demographic characteristics (occupation, long-term exposure to occupational or environmental agents, etc.), relevant medical history and symptom presentation.

## Data Availability

All pertinent data generated or analysed during this study will be included in the published article (and its supplementary information files). The audio used in the study will not be publicly available to protect participant privacy. An anonymous copy of the final (anonymised) datasets used and/or analysed during the current study will be available from the corresponding author on reasonable request upon approval of a proposal and with a signed data access agreement. Data will be made available for a specified research purpose to qualified external researchers whose proposed use of the data has been approved by their institutional review board. The request proposal must include a statistician. Data will be available beginning 6 months and ending 5 years following article publication. The full code and test sets will be made available on publication at the GitHub repository (https://github.com).

## Abbreviations

AI: Artificial intelligence
AV: Alveolar volume
AUROC: Area under the receiver operating characteristic curve
CAT: COPD assessment test
CI: Confidence intervals
COPD: Chronic obstructive pulmonary disease
CT: Computerised tomography
FEV_1_: Forced expiratory volume in 1 s
FCV: Forced vital capacity
FRC: Functional respiratory capacity
GDPR: General Data Protection Regulation
HRCT: High resolution chest CT
IIP: Idiopathic interstitial pneumonia
ILD: Interstitial lung disease
IPF: Idiopathic pulmonary fibrosis
K-BILD: King’s Brief Interstitial Lung Disease Questionnaire
K_CO_: Carbon monoxide transfer coefficient
LSTM: Lung short-term memory
LUS: Lung ultrasound
NSIP: Non‐specific interstitial pneumonia
STROBE: Strengthening the Reporting of Observational studies in Epidemiology
SVM: Support vector machine
TLC: Total lung capacity
TL_CO_: Transfer factor for carbon monoxide

## Footnotes

1 HRCT severity markers: traction bronchiectasis (3 levels); presence of honeycombing (3 levels); ground glass opacities (3 levels); reticulation (3 levels); and emphysema (3 levels). This analysis will be performed in all subjects with an available CT, and separately in IID and COPD patients and control subjects. Chest CT-scans will be reviewed independently by two radiologists blinded to each other.

2 Functional lung test severity markers: forced expiratory volume in 1 s (FEV_1_); forced vital capacity (FVC); FEV_1_/FVC; total lung capacity (TLC); functional respiratory capacity (FRC); transfer capacity for CO (TL_CO_]; K_CO_; alveolar volume (AV).
