# Supplementary material for: Deep learning diagnostic and severity-stratification for interstitial lung diseases and chronic obstructive pulmonary disease in digital lung auscultations and ultrasonography: clinical protocol for an observational case–control study
Source: BMC Pulm Med. 2023 Jun 2;23:191. doi: 10.1186/s12890-022-02255-w (PMC10234685; doi:10.1186/s12890-022-02255-w)
Supplement: Supplementary file 1 — Additional file 1. Self-administered questionnaire on demographic characteristics (occupation, long-term exposure to occupational or environmental agents, etc.), relevant medical history and symptom presentation. [file 12890_2022_2255_MOESM1_ESM.pdf]

DATA COLLECTION

We thank you for the time and effort you will take to complete this questionnaire. If you are unsure of what to write, please ask the study investigator or your regular doctor.

Study ID n° : ……………

**EXPOSURE TO TOBACCO / E-CIGARETTES / CANNABIS**		
1. Are you a current or former smoker of tobacco, e-cigarette or cannabis? *If YES, answer A-D. If NO, go to 2.*	☐ Yes	☐ No
A. Do you currently smoke regularly ? ______________________	☐ Yes	☐ No
B. At what age did you start smoking ? ______________________		
C. At what age did you quit smoking ? ________________ (If you are an active smoker, check NA)	☐ NA	
D. On average, how many cigarettes do you smoke per day ? ___________		
2. Have you been exposed to passive smoking ? *If YES, answer A-B. If NO, go to 3.*	☐ Yes	☐ No
A. Have you lived in the same house as someone who smokes ?	☐ Yes	☐ No
B. Have you been exposed to passive smoking (coffee shop, smoke-filled office?)	☐ Yes	☐ No

YOUR FAMILY'S RESPIRATORY HEALTH		
The following 3 questions concern people biologically related to you (parents, children, siblings)		
3. Has someone in your family suffered from a chronic respiratory disease (asthma, COPD, cystic fibrosis, bronchiectasis, lung cancer, ...) ?	☐ Yes	☐ No
4. Has anyone in your family had pulmonary fibrosis ?	☐ Yes	☐ No
5. Has anyone in your family suffered from autoimmune diseases (rheumatoid arthritis, scleroderma, ...) ?	☐ Yes	☐ No

LIVING ENVIRONMENT		
The following questions ask about your home or work/leisure environment. Answer "*yes*" if you were exposed regularly or repeatedly, especially in the 3 years before you started having respiratory problems.		
6. Jacuzzi / sauna / hot tub ?	☐ Yes	☐ No
7. Water leaks (washing machine, dishwasher, ...) or mold in your home ?	☐ Yes	☐ No
8. Down pillow/quilt (feather) ?	☐ Yes	☐ No
9. Birds (pigeons, chickens, canaries, ...) ?	☐ Yes	☐ No
10. Musty smell ?	☐ Yes	☐ No
11. Has your home suffered water damage ?	☐ Yes	☐ No
12. Do you have standing water in your house (aquarium, humidifier, aquatic plants, ...) ?	☐ Yes	☐ No
13. Do you regularly work at home with soil, compost or potted plants ?	☐ Yes	☐ No
14. Do you live on a farm ?	☐ Yes	☐ No

PROFESSIONAL LIFE AND HOBBIES

The following questions ask about activities (professions and hobbies) that you have done or are currently doing. Answer YES if you have done the activity even briefly.

15. Work with asbestos (insulation, roofing, ...) ?	☐ Yes	☐ No
16. Insulation of buildings/floors, installation of heaters, repair of roofs ?	☐ Yes	☐ No
17. Railway worker ?	☐ Yes	☐ No
18. Automotive mechanics (brake linings, clutches, seals, ...) ?	☐ Yes	☐ No
19. Sandblasting/Scraping ?	☐ Yes	☐ No
20. Plumbing/Tinsmithing ?	☐ Yes	☐ No
21. Working with talc ?	☐ Yes	☐ No
22. Working with beryllium ?	☐ Yes	☐ No
23. Working with aluminum ?	☐ Yes	☐ No
24. Work in the plastics industry?	☐ Yes	☐ No
25. Welder ?	☐ Yes	☐ No
26. Steel construction ?	☐ Yes	☐ No
27. Foundry ?	☐ Yes	☐ No
28. Agriculture ?	☐ Yes	☐ No
29. Road construction ?	☐ Yes	☐ No
30. Tunneling ?	☐ Yes	☐ No
31. Cement factory ?	☐ Yes	☐ No
32. Pottery ?	☐ Yes	☐ No

DRUGS AND TREATMENTS

The following questions are specific to the drugs and treatments you have received in your lifetime. Indicate if the treatment is current, past/completed or if you have never received it.

33. Amiodarone (Cordarone®)	☐ Current	☐ Past	☐ Never
34. Nitrofurantoin (Furadantine®, Uvamin®)	☐ Current	☐ Past	☐ Never
35. Methotrexate (Metoject®, Methotrexat®)	☐ Current	☐ Past	☐ Never
36. Radiotherapy on the thorax	☐ Current	☐ Past	☐ Never
37. Chemotherapy for cancer	☐ Current	☐ Past	☐ Never
38. Immunotherapy for cancer	☐ Current	☐ Past	☐ Never
39. Biological treatment (Rituximab, Tocilizumab...)<br>If yes, which one? ________________________________	☐ Current	☐ Past	☐ Never

SYMPTOMS AND HEALTH HISTORY

Your doctor will pay close attention to your respiratory symptoms during the visit. After the first 2 questions, the questionnaire asks about your non-respiratory symptoms and your health history.

BREATHING DIFFICULTIES

9. Have you ever experienced breathing difficulties (shortness of breath, difficulty breathing) beyond what is normally expected during exercise?

☐ Yes ☐ No

If "Yes" A. When did the difficulties arise? Month (mm):___________________Year (20xx):_______

B. Place a mark on the line below that corresponds to the current intensity of your breathing difficulties (shortness of breath, difficulty breathing)

|____|

No difficulty in breathing — Unbearable difficulty to breathe

COUGH

10. Do you cough regularly? ☐ Yes ☐ No

(With or without secretions/sputum/clots/mucus/clutter)

If "Yes" A. When did the cough start ? Month (mm):_________________ Year (20xx):_____________

B. Place a mark on the line below that corresponds to the current intensity of your cough

|____|

No cough — Unbearable cough

C. Is your cough accompanied by secretions? ☐ Yes ☐ No

SYMPTÔMES NON RESPIRATOIRES

11. Answer "Yes" if you regularly experience any of the symptoms listed below

A. Fatigue	☐ Yes	☐ No
B. Stiffness (>1 hour in the morning) or joint pain or swelling<br>*If "Yes"* What articulations:<br>☐ Hands/wrists ☐ Shoulders<br>☐ Knees ☐ Ankles/feet	☐ Yes	☐ No
C. Difficulty swallowing or feeling of food stuck in the throat ?	☐ Yes	☐ No
D. Permanent dryness of the eyes and mouth ?	☐ Yes	☐ No
E. Pain or change in color (red, white, bluish) of fingers exposed to cold (Raynaud's phenomenon)<br>*If "Yes", starting age*:__________	☐ Yes	☐ No
F. Weight loss of more than 5 kg in 6 months ?	☐ Yes	☐ No
G. Gastric burning or acidic, unpleasant mouth taste after eating ?	☐ Yes	☐ No
H. Rashes on the skin (red spots) ?	☐ Yes	☐ No
I. Muscle weakness (new difficulty getting up from a chair, carrying a heavy object)	☐ Yes	☐ No

HEALTH HISTORY

12. The following questions ask about your health history. Answer "yes" if a doctor has told you at least once that you have this problem

A. Asthma	☐ Yes	☐ No
B. COPD	☐ Yes	☐ No
C. Lung cancer	☐ Yes	☐ No
D. Tuberculosis	☐ Yes	☐ No
E. Heart failure, myocardial infarction, angor, coronary artery disease	☐ Yes	☐ No
F. Diabetes	☐ Yes	☐ No
G. Rheumatoid arthritis	☐ Yes	☐ No
H. Scleroderma	☐ Yes	☐ No
I. Lupus erythematosus	☐ Yes	☐ No
J. Polymyositis or dermatomyositis or anti-synthetase syndrome	☐ Yes	☐ No
K. Sjoegren's syndrome	☐ Yes	☐ No
L. Gastroesophageal reflux or hiatal hernia	☐ Yes	☐ No
M. Sleep apnoea syndrome	☐ Yes	☐ No
N. Immune deficiency or immunodeficiency (lack of antibodies)	☐ Yes	☐ No
O. Other autoimmune disease: ______________________________		

We are very grateful for your time and effort in completing this survey.

Note: This questionnaire is largely based on one administered at the Providence Health Care Centre, St. Paul's Hospital, University of British Columbia, Vancouver, BC. The Respiratory Department at St. Paul's Hospital (Prof Chris Ryerson) agreed to its translation into French and its use in the pulmonology department at the Centre Hospitalier du Valais Romand (CHVR) and at the Hôpital Riviera Chablais (HRC). French adaptation: P-O. Bridevaux, April 2022. All rights reserved.
